# Scientific Discoveries Supporting Theories in Science: From Thinking to Practice

**DOI:** 10.3390/ijms232315025

**Published:** 2022-11-30

**Authors:** Stefano Fais

**Affiliations:** Department of Oncology and Molecular Medicine, Istituto Superiore di Sanità, 00161 Rome, Italy; stefano.fais@iss.it

The idea to propose this ambitious title for a Special Issue in the *International Journal of Molecular Science* came, on one hand, from my personal experience in research in medicine, lasting 41 years, which has often been inspired by chance. However, on the other hand, the history of medicine is characterized by key discoveries that have been generated from apparently failed experiments. Therefore, while it is mandatory that science always needs a rigorous project-oriented spirit, at the same time, a sacral respect for chance and new roads should coexist. In fact, science needs daily activities, open to a continuous mutability, in order to remain open to new horizons, moment by moment.

For the above reasons, I want to start from an article published in the *Financial Times*, in 2008. It has an apparently provocative title: “Drug research needs serendipity” [1]. In this visionary article, the authors state that, in the last two decades, not too many, if not only few, new and effective drugs have been introduced into the market. This is despite the huge investments and they tried to propose some explanation for this. In their own words: “*What went wrong? The answer, we suggest, is the mis-measure of uncertainty, as academic researchers underestimated the fragility of their scientific knowledge, while pharmaceuticals executives overestimated their ability to domesticate scientific research*.” and “*Medical research is particularly hampered by the scarcity of good animal models for most human disease, as well as by the tendency of academic science to focus on the “bits and pieces” of life–DNA, proteins, cultured cells–rather than on the integrative analysis of entire organisms, which can be more difficult to study*.” A paradox of this low effectiveness of the new drugs is that a new field in pharmacology was born, whose goal is to discover the off-targeting of known drugs through their side effects [2]. However, this is not surprising, as the vast majority of drugs that pioneered the pharmacology of neurologic diseases was envisioned for other uses [3]. Actually, in the past, serendipity had a key role in epochal discoveries and was a fairly common occurrence in science. One example from the past is the discovery of penicillin. Fleming was studying *Staphylococcus aureus* as a possible main cause of influenza. One of his culture plates became contaminated by a mold that created a bacteria-free circle. He observed that the mold inhibited the growth of bacteria and, from this simple observation, he found within the mold a substance that was very active against the vast majority of the bacteria infecting human beings, which he called *Penicillin* [4]. One other example, while far less known, is that of the 1931 Nobel Prize winner Otto H. Warburg. He left some plates containing tumor cells seeded in a culture medium in the laboratory’s incubator overnight with the usual 37 °C and O_2_/CO_2_ atmosphere. The morning after, he realized that the O_2_ dropped down within the incubator, expecting to find all the cells dead due to the hypoxic conditions. However, the cells were in perfect health and, after his initial astonishment, he thought that cancer cells likely did not need oxygen to live. After a series of experiments, his conclusion was that, differently to normal cells, cancer cells do not need oxygen for their metabolism, while they ferment sugar, in turn, producing lactate, that contributed to extracellular acidification by releasing H+, thus, laying the foundations for subsequent studies on the role of extracellular acidity in tumor pathogenesis and malignant progression [5,6].

All in all, these considerations convinced me that some steps back should be taken in science, with the aim to rediscover the role of chance in scientific research [7,8]. Therefore, we should identify serendipity as part of the scientific process, suggesting that “unexpected discoveries” should become part of life science. Serendipity should be considered an essential part of the scientific method and, particularly, a tool for progress, and it should be taken as a rational approach to scientific practice—an attitude and a happy accident. We should not think of serendipity as merely luck, chance, or happenstance, but instead as a process in which a fortunate event leads to the discovery of a new, unexpected solution to a problem [7,8].

We had an impressive consensus, with up to 20 articles published, with a broad level of interest for readers.

The Issue includes four articles dealing with various aspects of **metabolism.** A very challenging review [9] proposed the shift from catabolism to anabolism, also known as the Warburg effect, as a common metabolic feature of many diseases, in turn, suggesting that a careful look should be directed to old drugs in a way that they should be repurposed.

A very interesting scientific article showed that the measurement of serum [Cl^−^] may represent an easy and useful marker, helping to estimate metabolic condition [10].

Another article in the area of metabolism face-off is the problem of obesity-related insulin resistance by evaluating the expression of a new glucose transporter, SLC2A6 (GLUT6) [11]. They found that GLUT6 is markedly upregulated in pancreatic islets from genetically obese leptin-mutant (ob/ob) and leptin receptor-mutant (db/db) mice, compared to lean controls. The authors showed that GLUT6 knockdown mice secreted more insulin in response to high-dose glucose, compared to wild-type mice for GLUT6, with no adverse impact on body mass, body composition, or glucose tolerance. This study demonstrates that GLUT6 plays a role in pancreatic islet insulin secretion in vitro but it is not a dominant glucose transporter that alters whole-body metabolic physiology in ob/ob mice.

Lastly, a study focusing on metabolism deals with the peripheral blood lymphocyte response to a dose of γ-rays in patients treated with radioiodine (I-131) for hyperthyroidism as compared to healthy individuals [12]. Phosphorylated histone variant H2AX (γ-H2AX) and micronuclei (MN) induction were used to determine the change in PBL radio-sensitivity and the correlations between the two types of damage. Actually, the two assays showed large inter-individual variability, suggesting that PBL radio-sensitivity should not be considered a general criterion to establish γ-ray-induced damage.

One scientific article and a review introduced the fascinating issue of **fruit nanovesicles**. Plant-derived nanovesicles (PDNVs) have recently received great attention regarding their natural ability to deliver several active biomolecules and antioxidants.

The scientific report investigated the bioactive cargos of nanovesicles from different fruits, comparing fruits derived from organic farming to the same fruits produced by conventional agriculture [13], showing that, at equal volume, nanovesicles from organic-farming-derived fruits were greater, in terms of absolute number, and with higher antioxidant activity compared to nanovesicles obtained from conventional agriculture-derived nanovesicles. Moreover, the authors set up mixes of different fruits with comparable levels of ascorbic acid, catalase, glutathione, and superoxide dismutase 1. Finally, the authors exposed fruit nanovesicle mixes to either chemical or physical lytic treatments, but no effects were observed on the number, size, and antioxidant capacity of the treated nanovesicles, thus, showing a marked resistance of PDNVs to external stimuli and a high capability to preserve their content. This, of course, supported the use of organic-agriculture-derived fruits as the most suitable source for nanovesicles that, for their content, may represent an ideal source of anti-oxidant supplementation for human beings.

A review introduced an important and, for many reasons, unmet issue in the complex world of extracellular vesicles: the source of EVs, including the cells and the species from which Evs are obtained and the microenvironmental condition during EV production [14]. This issue is of particular interest when the choice of the most suitable EVs for drug delivery is the central problem. In fact, the use of EVs of human origin has at least two major problems: (i) autologous EVs from a patient may deliver dangerous molecules; and (ii) the production of EVs is not suitable for large-scale industrial use. Existing studies suggest that plant-derived nanovesicles (PDNVs) may represent a valuable tool for extensive use in health care.

A series of articles tackled the issue of **cancer** pathogenesis and potential therapies. The review from Koltai T et al. [15] dealt with the role of the cell surface protein CD44, the main receptor for the binding of hyaluronan, in cancer growth and progression, with a particular focus on pancreatic ductal adenocarcinoma. They start from the evidence that malignant tumors show over-expression/over-activity of both CD44 and hyaluronan and, also, from the evidence for independent inhibition of hyaluronan-producing cells, hyaluronan synthesis, and/or CD44 expression, which has been found to decrease the tumor cell’s proliferation, motility, invasion, and metastasis. For non-small-cell lung cancer (NSCLC), they suggest that combination therapy, including the above compounds, may represent a future approach against cancer and, in particular, pancreatic cancer.

Two articles deal with non-small-cell lung cancer (NSCLC). The article by Fanini F. et al. [16] explores the possibility of using treatment with the tumor suppressor miR-16 to restore tyrosine kinase inhibitor (TKI) sensitivity in a model of KRAS-mutated non-small-cell lung cancer (NSCLC) cells. They performed both in vitro and in vivo experiments, showing that miR-16 restored the sensitivity to erlotinib in KRAS-mutated NSCLC, directly targeting the three KRAS downstream effectors MAPK3, MAP2K1, and CRAF, supporting the use of miR-16 in combination with erlotinib in the treatment of NSCLC with KRAS mutation.

Madsen, K.L. et al. [17] investigated a new approach to overcome resistance of cancer stem cells (CSCs) derived from NSCLC patients to chemotherapy. For this purpose, they sorted primary patient-derived NSCLC cells based on their expression of the CSC marker CD44, in turn, investigating the effects of cisplatin and a thymidine analog (deoxyuridine) labeled with an Auger electron emitter (125I) against these cells. The results showed that while the CD44+ populations are more resistant to cisplatin than the CD44− populations, the labelling with the thymidine analog 5-[125I]iodo-20 -deoxyuridine ([125I]I-UdR) proved to be equally effective against CD44− and CD44+ populations, suggesting that Auger electron emitters may be efficiently used in resistant lung cancer CD44+ populations as well.

The article by Koirala, N. et al. [18] reviews the possibility to implement therapies with CDK 4/6 inhibitors (palbociclib, ribociclib, and abemaciclib) with HER2-directed therapies for (ER+/−) HER2+ breast cancer patients with evidence of metastasis. Some clinical studies, that would like to replace traditional chemotherapy, support this hypothesis.

The article by Sauer, N et al. [19] reviews the evidence that targeting the LAG-3 (Lymphocyte activation gene 3) protein may represent a valuable new anti-cancer approach. LAG-3 is considered an immune escape factor of tumors that is diffusely expressed from tumor cells. A series of LAG-3 inhibitors has been tested in clinical trials and this review critically reviews the obtained results, suggesting some options for the future.

The last article in the cancer series is from Musso, N et al. [20]. It deals with the potential application of Dielectrophoresis (DEP) in patients with Multiple Myeloma (MM). The background of this review is the problem of characterizing circulating tumor plasma cells (CTPCs) in MM patients. DEP is an emerging label-free cell-manipulation technique to separate cancer cells from healthy cells in samples of peripheral blood mononuclear cells. The idea was to separate normal and MM-related cells, based on phenotype and membrane capacitance. They summarize some preclinical data on the use of DEP for CTPC detection, emphasizing some potential for the future.

A further series of articles involves **other diseases** as well, including Atrial Fibrillation, Spinal Muscular Atrophy, and Duchenne Muscular Dystrophy, an X-linked recessive disorder. Two articles present data on different molecular mechanisms involved in atrial fibrillation. The first article, by Thibault, S. et al. [21], provides data supporting sex differences in susceptibility to atrial fibrillation. It begins from the epidemiological evidence that males have a higher risk of developing atrial fibrillation (AF) than women. Inasmuch as electrophysiological studies in atrial myocytes did not show significant differences between males and females, the other moved on investigation on the role of connexins lateralization in this disease. They showed that male atrial myocytes had more lateralization of connexins as compared to female mice, and this was consistent with a larger atria and atrial myocyte than females and the fact that orchiectomy reduced AF susceptibility in males by decreasing connexin lateralization and atrial myocyte size, thus, supporting a role for androgens in this pathological condition. The other article [22] explores the same male predisposition to AF by studying sex differences in intracellular Ca^2+^ homeostasis in mouse atrial myocytes. They used programmed electrical stimulation (EPS) protocols, showing that the Ca^2+^ transient amplitude was higher in male atrial myocytes together with the Na^+^ -Ca^2+^ exchanger (NCX1) current density, resulting in more spontaneous systolic and diastolic Ca^2+^ releases, supporting a further risk factor in males for AF.

The other two articles reported data on rare diseases. The first, from Studzi’nska, S et al. [23], investigated the role of treatment with oligonucleotides in spinal muscular atrophy, a genetic disease. They used nusinersen oligonucleotide to detect both nusinersen and its metabolite in the serum of treated individuals. With an innovative method, both nusinersen and a dozen of its metabolites were successfully identified in the serum samples. The other study [24] was carried out on a patient with Duchenne muscular dystrophy (DMD), an X-linked recessive disorder. This disease mostly affects males but the authors show a case report of a female affected by DMD, where the disease was due to a balanced X-autosome reciprocal translocation that disrupts the DMD gene that, together with the skewed X-inactivation, induced the manifestation of the DMD phenotype.

A series of further articles involves a more **patho-physiological area**. A first one, by Pesce, M et al. [25], is a review dealing with bacterial engineering in order to genetically modify probiotics, representing a new biotechnological approach in the treatment of Inflammatory Bowel Diseases (IBDs) that includes both Ulcerative Colitis and Crohn’s Disease. These diseases are commonly treated with corticosteroids and immunosuppressive drugs, too often as a life-long treatment, with a high level of side effects and a clear exposure to the risk of relevant long-term side effects. For this reason, engineered probiotics represent a revolutionary and, for many reasons, mandatory therapy in IBD.

The article by Detzner, J. et al. [26] is a review that discusses the clinical impact of infections by Enterohemorrhagic *Escherichia coli* (EHEC), a human pathogenic subset of Shiga toxin (Stx)-producing *E. coli* (STEC). It is an important issue inasmuch as Stx, together with localizing through a vesicular package in the intestine, travels through the body into the blood stream, in turn, being the major cause of Stx-mediated extraintestinal complications. The authors summarize data on Stx cellular targets and also the low susceptibility of primary renal and colonic epithelial cells that suggest a high level of resilience of these epithelia to the pathogenicity of Stx subtypes.

Zhang, Y. et al. [27] showed results on a new approach to avoid the invasive procedure of liver biopsies in patients affected by liver injuries, in particular, in the condition of non-alcoholic fatty liver disease (NAFLD), which affects an extensive number of human beings worldwide. For this reason, they used hepatic and serum proteomic analysis based on samples from leptin-receptor-deficient mice (db/db), a diabetic mouse model affected by obesity and NAFLD. The authors investigated a series of potential serum biomarkers to be used as a non-invasive approach in this pathology, identifying some Differential Expressed Proteins (DEPs) involved in many cellular pathways, together with a different expression of DEPs between the liver and serum.

The article by Tessema, B et al. [28] presents data on factors affecting the cellular stress assay (CSA) in peripheral blood mononuclear cells (PBMCs) as potential biomarkers for many diseases’ progression. They show that the CSA parameters measured in PBMCs are influenced by the PBMC isolation method, age, seasonal variation, and gender, suggesting one should take care of these factors in investigating human PBMCs. Most of all, in assessing the cellular stress in PBMCs, both extracellular acidification and mitochondrial respiration should be taken into careful account.

I consider this series of articles very innovative and challenging, making this Special Issue very successful and, in turn, convincing us to propose a new 2.0 Issue that is starting in a very promising way.

A last comment I would like to make is that molecular science is very important and innovative, and when it is aimed not only to increase our knowledge but most importantly to improve the health of human beings, it should take into careful account that any kind of potential preventive approach or therapy is directed to the whole body and our body is a very complex and integrated organism that is in continuous connection. Finally, I direct a recent perspective article proposing a new deal in drug discovery by moving the target of future therapies from the cell to the microenvironment [29].
